# Health insurance and end-of-life healthcare expenditures: evidence from Chinese Longitudinal Healthy Longevity Survey

**DOI:** 10.1038/s44401-026-00084-1

**Published:** 2026-04-27

**Authors:** Liangjun Song, Xiaochen Zhang, Mingqi Wang

**Affiliations:** 1 Fudan Development Institute, Fudan University, Shanghai, China; 2 Division of Social Sciences, Duke Kunshan University, Kunshan, China; 3 Global Health Research Center, Duke Kunshan University, Kunshan, China; 4 Actuarial Department, Taiping Life Insurance Co., Ltd, Shanghai, China

**Keywords:** Health care, Medical research

## Abstract

The terminal stage of life accounts for a substantial share of healthcare expenditures over the life course. Using data from the Chinese Longitudinal Healthy Longevity Survey (2005–2018), this study examines the impact of health insurance coverage on healthcare spending during the last year of life. To address potential selection bias, we first estimate the probability of insurance coverage using logistic regression and then compare expenditure patterns between insured and uninsured populations through propensity score matching. We further conduct rural–urban and regional subgroup analyses to explore heterogeneity in insurance effects. Our results reveal significant differences in medical expenditures associated with insurance utilization, while no significant disparities are observed in nursing care costs across groups. These findings provide new evidence about increasing healthcare spending in a non-Western context, and call for a more balanced health system to reduce inequalities in end-of-life healthcare due to insurance coverage among the elderly.

## Introduction

China has the world’s largest size of elderly population. In the era of population aging, life expectancy at the age of 60 almost doubled in China, from 11.0 to 19.2 years during the period 1950–2015, increasing the size of population above 60 years old to a striking 310 million in 2024^1^. In the meantime, death comes later in life for many and dying is often prolonged^2^. As a result of these demographic changes, there still exists a gap between life expectancy and healthy life expectancy. Existing evidence indicates that health status deteriorates rapidly in the period immediately preceding death^3^. As a result, the bulk of end-of-life healthcare expenditures is concentrated in periods of severe health decline, which coincide with the widening gap between life expectancy and healthy life expectancy^4^. Thus, there has been a rising concern about the potential escalation of healthcare costs in the future^5^. However, such concerns are mitigated by the “red herring hypothesis”, which attributes the increased healthcare expenditure to proximity to death rather than age itself^6,7^. Specifically, there is a substantially greater magnitude of expenses incurred prior to death compared to earlier life stages^8^. For example, previous studies found that the healthcare expenditure during the last year of life accounts for 25–30% of total lifetime medical costs^9–11^. The average annual medical expenditure for patients aged 65 and older in their final year of life is approximately five times higher than for non-terminal patients^12^. Hence, the vast majority of healthcare expenditures is associated with the terminal phase of their life cycle rather than driven by age per se^13^. Several reforms in the health systems, such as hospice services or skilled nursing facilities, have been proposed to mitigate the rising healthcare expenses at the end of life. However, evidence regarding the efficacy of these approaches remains inconclusive^14,15^.

Health insurance serves as the primary means of financing healthcare expenses, especially for those who might otherwise find it challenging to afford essential medical services. While insurance status does not guarantee adequate access to healthcare, it typically lowers the financial barriers, expands the range of available providers and services, and reduces out-of-pocket uncertainty^16^. However, like other forms of insurance, health insurance is inherently affected by issues such as moral hazard and adverse selection. For instance, beneficiaries may have strong incentives to engage in fraudulent activities during the reimbursement process, and health insurance companies may encounter challenges in detecting pre-existing conditions^17^. Among the factors influencing healthcare expenditure, health insurance receives significant attention from academia. Evidence from both developed and developing countries has shown that individuals with health insurance exhibit significantly higher utilization of healthcare services^18,19^.

Within the context of end-of-life, health systems confront two core challenges: disparities in insurance access and mounting pressure for cost containment. On one hand, some people are overtreated at the end of life despite of costly treatment, while others are undertreated dying of preventable conditions and witout basic pain reliefs^2^. Such inequality in death and dying has been amplified by the unbalanced insurance coverage^20^. On the other hand, access to health insurance significantly adds to the growth of healthcare expenditure, resulting from issues like adverse selection and moral hazard, which is especially severe at the end of life. The presence of uncertainty in health risks complicates the accurate forecasting of future expenditures and the design of sustainable health systems^21^, contributing to the massive pressure on cost containment. Thus, understanding the relationship between insurance coverage and end-of-lfie healthcare expenditure becomes critical in designing a better health system for death and dying.

We have chosen China’s data to investigate the insurance effects on healthcare expenditures at the end of life mainly for two reasons. Firstly, factors such as rural-urban disparities are distinctive to the Chinese context^22,23^. As we delve deeper into China’s case, it becomes evident that the rural-urban divide in China is not just a matter of geography. It is also reflected in the disparities in healthcare access and quality. In rural areas, residents often face higher out-of-pocket costs due to the lack of affordable and accessible healthcare facilities, as well as the need to travel longer distances to reach specialized care. On the other hand, urban areas tend to have more advanced and specialized healthcare facilities. However, this also means that urban residents often face higher costs for advanced services^24^. The major insurance schemes administered by the Chinese government, while improving access, have not fully addressed these disparities^25^. Secondly, while prior research has documented diverse patterns in healthcare utilization across developed countries^26^, the lack of understanding beyond western countries presents a significant limitation. This oversight can lead to issues of over-generalization, given that perceptions of a “good death” can vary greatly among different cultures^27^. It is imperative to recognize that those factors that define the concept of a “good death” can shape the patterns of healthcare expenditure at the end of life^28^. These defining characteristics, such as being a burden to others and avoidance of futile life-prolonging interventions, are deeply rooted in a country’s history and cultural values^29^. Therefore, evidences from countries with distinct cultural and institutional backgrounds can provide valuable insights into how these factors shape end-of-life experiences.

Building on previous research that explores the relationship between healthcare expenditure and health insurance, our study delves into the primary factors shaping healthcare expenditure during the last year of life for elderly individuals in China, and explores whether there exists discernible spending patterns among the insured and uninsured population. Our research makes at least three key contributions to the existing literature. Firstly, we find having insurance significantly increases end-of-life spending on medical service, but no such effects are found on nursing care. Such disparities indicate another layer of inequality that exists in insurance coverage on long term care. Secondly, we have directed our attention towards healthcare expenditures incurred during the final year of life. Our findings contribute to the ongoing discussion about the relationship between proximity to death and the elevated costs. Thirdly, we have conducted our analysis using longitudinal survey data from China, where the elements of “good death” can be vastly different from western countries and thereby shape unique end-of-life spending patterns.

## Results

### Multivariate regression

Our empirical results are reported in Table 1. In Column (1), Ordinary Least Square (OLS) regression results are presented. Individuals whose medical expenses were predominantly covered by health insurance spent 16.5% more during their final year of life (significant at *p* < 5%). Additionally, we applied the Tobit model to address 866 left-censored and 95 right-censored observations, with the outcomes reported in column (2). Furthermore, to investigate potential variations in the effects of health insurance coverage, we analyzed determinants across different expenditure quantiles. Columns (3), (4), and (5) demonstrate consistent effects, with increases ranging from 22.6% (significant at *p* < 5%) in the 25th quantile to 34.9% (significant at *p* < 1%) in the 75th quantile. These findings confirm the heightened utilization of healthcare services attributable to health insurance access.

**Table 1:** Results of multivariate regression

	(1)	(2)	(3)	(4)	(5)
	OLS	Tobit	0.25 Quantile	0.5 Quantile	0.75 Quantile
inspay	0.165**	0.161*	0.226**	0.304***	0.349***
	(0.0804)	(0.0829)	(0.111)	(0.0626)	(0.0670)
age	−0.0445***	−0.0478***	−0.0452***	−0.0354***	−0.0344***
	(0.00329)	(0.00378)	(0.00399)	(0.00268)	(0.00290)
female	−0.0292	−0.0266	0.0356	0.00700	−0.104*
	(0.0641)	(0.0732)	(0.0688)	(0.0502)	(0.0540)
cohabit	0.443***	0.495***	0.328***	0.460***	0.427***
	(0.0820)	(0.0924)	(0.105)	(0.0676)	(0.0670)
city	0.293***	0.314***	0.163**	0.321***	0.303***
	(0.0621)	(0.0688)	(0.0778)	(0.0477)	(0.0532)
income	0.117***	0.130***	0.192***	0.0701***	0.0777***
	(0.0155)	(0.0152)	(0.0384)	(0.0124)	(0.0106)
schooling	0.150***	0.175***	0.138***	0.147***	0.123***
	(0.0387)	(0.0476)	(0.0440)	(0.0290)	(0.0353)
qolind	0.156***	0.172***	0.155***	0.111***	0.100***
	(0.00684)	(0.00714)	(0.00917)	(0.00566)	(0.00594)
pain	−0.286***	−0.319***	−0.285***	−0.199***	−0.143***
	(0.0265)	(0.0311)	(0.0334)	(0.0230)	(0.0231)
dhospital	1.348***	1.487***	1.267***	1.006***	0.829***
	(0.0888)	(0.120)	(0.116)	(0.0764)	(0.0828)
Year FE	YES	YES	YES	YES	YES
Province FE	YES	YES	YES	YES	YES
cons	8.791***	8.782***	7.812***	9.023***	10.28***
	(0.442)	(0.479)	(0.568)	(0.345)	(0.361)
sigma		2.825***			
cons		(0.0242)			
*N*	8339	8339	8339	8339	8339
R-sq	0.192				

Standard errors are reported in the brackets. *p < 0.1. **p < 0.05. ***p < 0.01.

Regarding the disparity between rural and urban areas, our findings indicate that residing in urban areas tends to result in a significantly higher level of medical consumption, achieving statistical significance at a level of *p* < 1% in the majority of instances. This finding underscores the profound impact of geographic location on health service usage patterns, with the convenience, accessibility, and greater availability of healthcare facilities in urban centers likely contributing to this observed disparity in medical consumption.

We discovered that older adults residing in urban settings, cohabiting with a partner, and self-reported pain exert a more substantial influence in the upper quantiles, whereas incomes have a comparatively minor impact. Remarkably, gender generally fails to significantly affect health expenditure. Furthermore, one’s health status profoundly influences expenditures on medical services. Elderly individuals with lower scores in activities of daily living (ADL) and severe pain tend to incur higher costs. To further investigate potential discrepancies between the insured and uninsured populations, we examined the coefficients of explanatory variables for these two groups in Table 2. Among the insured group, all covariates were statistically significant. However, among the uninsured, we observed that gender and education attainment had little to no significant effect on medical expenditures.

**Table 2:** Results of multivariate regression based on different groups

	(1)	(2)	(3)	(4)
	OLS insured	OLS uninsured	Tobit insured	Tobit uninsured
main				
age	−0.0532***	−0.0424***	−0.0610***	−0.0448***
	(0.00736)	(0.00369)	(0.00861)	(0.00418)
female	−0.298**	0.0451	−0.310*	0.0454
	(0.141)	(0.0722)	(0.163)	(0.0819)
cohabit	0.327**	0.483***	0.401**	0.523***
	(0.162)	(0.0952)	(0.188)	(0.106)
city	0.448***	0.236***	0.506***	0.247***
	(0.146)	(0.0683)	(0.163)	(0.0752)
income	0.105***	0.118***	0.119***	0.130***
	(0.0292)	(0.0183)	(0.0308)	(0.0175)
schooling	0.226***	0.0689	0.280***	0.0696
	(0.0620)	(0.0500)	(0.0831)	(0.0601)
qolind	0.159***	0.154***	0.183***	0.168***
	(0.0153)	(0.00755)	(0.0163)	(0.00789)
pain	−0.252***	−0.305***	−0.283***	−0.337***
	(0.0549)	(0.0303)	(0.0669)	(0.0350)
dhospital	1.177***	1.352***	1.361***	1.441***
	(0.145)	(0.118)	(0.206)	(0.155)
cons	9.745***	8.563***	9.924***	8.512***
	(0.877)	(0.546)	(1.024)	(0.554)
Year FE	YES	YES	YES	YES
Province FE	YES	YES	YES	YES
sigma				
cons			3.138***	2.706***
			(0.0565)	(0.0264)
*N*	1986	6353	1986	6353
R-sq	0.231	0.173		

Standard errors are reported in the brackets. *p < 0.1. **p < 0.05. ***p < 0.01.

### Propensity score matching

Before implementing the matching procedure, we first examined the socioeconomic and demographic factors associated with health insurance enrollment by estimating a logistic regression model for the propensity score. The results in Table 3 reveal a clear pattern of selection into insurance. Urban residence (*β* = 0.661, *p* < 0.01), higher household income (*β* = 0.048, *p* < 0.01), formal employment (*β* = 0.984, *p* < 0.01), and higher education (*β* = 0.195, *p* < 0.01) were all strong positive predictors of insurance coverage. Conversely, reporting worse health status (pain) was associated with a lower probability of being insured (*β* = −0.097, *p* < 0.01). These findings indicate that insurance access among older adults in China is systematically correlated with socioeconomic advantage and may present barriers to those in greater need. The coefficients for survey year dummies also show a significantly positive trend over time, corroborating the national expansion of insurance coverage during the study period. This model was used to generate a propensity score for each individual, reflecting their predicted probability of being insured conditional on observed characteristics.

**Table 3:** Results of logistic regressions (determinants of health insurance enrollment)

Variable	Coefficient	(S.E.)	*p*-value
Urban Residence (city)	0.661***	−0.06	<0.001
Log Income (lninc)	0.048***	−0.013	<0.001
Office Job	0.984***	−0.127	<0.001
Years of Schooling	0.195***	−0.044	<0.001
Pain Level	−0.097***	−0.028	<0.001
Survey Year Trend	Controlled (positive and increasing)		
*N*	8339		

***p < 0.01. All estimates are statistically significant at the 1% level.

**Fig. 1:**
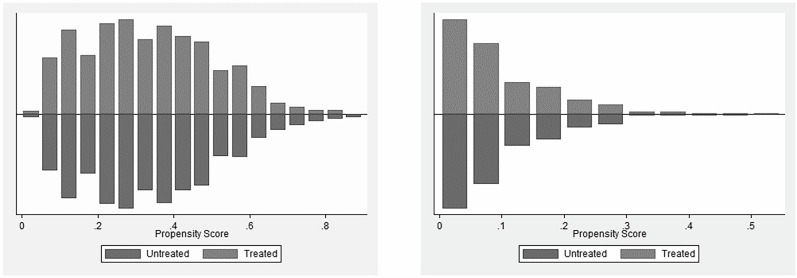
Post-matching analysis of propensity score distributions. This figure presents the distribution of propensity scores for treated and untreated groups after matching. The left panel corresponds to the treatment definition used in Columns (1) (2) of Table 4 (“*Inspay*”), and the right panel corresponds to the treatment definition used in Columns (3) (4) of Table 4 (“*Inspaycare*”). In each panel, bars above the horizontal axis represent treated observations and bars below the axis represent untreated observations.

In total, we successfully matched 1794 out of 1986 observations in our sample. The comparison results are reported in Fig. 1. The histograms illustrated that the propensity scores of the two groups were effectively balanced after matching. Specifically, we hypothesized that health insurance coverage could be predicted by specific factors, and subsequently conducted a regression analysis to evaluate the role of these factors in determining the probability of coverage. The estimated coefficients were then utilized to compute a score for each respondent, enabling us to match individuals with similar scores.

Table 4 reports the average treatment effects of having access to health insurance. After matching, the medical costs for health insurance beneficiaries were on average 2232.82 RMB higher than those without health insurance. Note that place of residence (people who residence in urban areas) has been controlled and matched. In Column (2), nursing care costs between the two groups were compared, revealing no significant effects of health insurance on nursing care spending, which is plausible given that most health insurance in China does not cover nursing care services. Regarding care costs, there was a specific question identifying the major source of payment for care costs. We constructed the variable *inspaycare* in a similar way as we did to the *inspay* variable. After dropping all missing values, we still have 7473 individuals in the sample, and the treated group “care insured group” has 324 participants. In Column (3) medical costs in the treated and control groups were compared, indicating that the insured group spent 3450 RMB more than the uninsured. Additionally, Column (4) found no significant difference in nursing care costs between the two groups.

**Table 4:** Average treatment effects using propensity score matching

	(1)	(2)	(3)	(4)
Treatment	*Inspay*	*Inspay*	*Inspaycare*	*Inspaycare*
Outcome variable	Medical costs (*N* = 1794)	Nursing Care costs (*N* = 1748)	Medical costs (*N* = 322)	Nursing Care costs (*N* = 322)
Treated	10267.8155	6712.50801	17369.9464	6648.85714
Control	8034.99777	7626.78833	13919.4669	6677.3323
Difference	2232.81773	−914.28032	3450.4795	−28.4751553
S.E.	568.671882	614.671153	2035.35481	1237.04966
T-statistic	3.93	−1.49	1.70	−0.02

Table 5 demonstrated that the matching procedure was highly effective in creating comparable treatment and control groups. After matching, the standardized mean differences (SMDs) for all covariates were reduced below 5%, well under the recommended threshold of 10%. The median absolute bias was reduced from 18.6 to 1.3%. Furthermore, the joint insignificance of all covariates post-matching is confirmed by a likelihood ratio test (*p* = 0.997), and the pseudo-*R*² is negligible (0.002). These metrics collectively indicate that the matched groups are excellently balanced on all observed pre-treatment characteristics.

**Table 5:** Balance of covariates before and after propensity score matching

	Unmatched Samples		Matched Samples		% Bias Reduction	SMD After Matching
Covariate	Mean Treated	Mean Control	Mean Treated	Mean Control		
Demographics						
age	92.15	94.52	92.64	92.46	92.40%	1.90%
female	0.45	0.62	0.49	0.5	90.30%	3.30%
Socioeconomic						
city	0.59	0.37	0.55	0.55	99.80%	0.10%
schooling	0.71	0.35	0.58	0.57	98.00%	0.90%
income	9.57	9.17	9.49	9.4	76.30%	4.20%
Health Status						
pain	3.52	3.68	3.55	3.57	91.70%	1.20%
qolind	13.12	13.05	13.14	13.17	61.00%	0.60%
dhospital	0.2	0.06	0.15	0.14	92.00%	3.60%
Summary Statistics						
Mean Absolute Bias	20.00%		1.70%			
Median Absolute Bias	18.60%		1.30%			
Pseudo *R*²	0.162		0.002			
LR Chi-sq (*p*-value)	0		0.997			

To test the robustness of our findings, we employed multiple alternative propensity score matching (PSM) strategies. As shown in Table 6, the estimated Average Treatment Effect on the Treated (ATT) for medical expenditures remains positive, statistically significant at the 1% level, and within a consistent range of 2085–2576 RMB across all specifications. This includes *k*-nearest neighbor matching (with *k* = 3 and *k* = 5), kernel matching, and variations in caliper width (0.01 and 0.1). The remarkable consistency of these results strongly confirms the robustness of our main conclusion that health insurance significantly increases medical expenditure at the end of life.

**Table 6:** Estimates of average treatment effect on the treated (ATT) using alternative matching strategies

Matching Method	ATT	(S.E.)	[t-stat]
1:1 Nearest Neighbor (Caliper = 0.05)	2232.82***	−568.67	[3.93]
K-Nearest Neighbor (*k* = 3)	2461.60***	−588.14	[4.19]
K-Nearest Neighbor (*k* = 5)	2443.70***	−560.58	[4.36]
Kernel Matching	2576.35***	−516.22	[4.99]
1:1 NN with Narrow Caliper (0.01)	2277.39***	−567.34	[4.01]
1:1 NN with Wide Caliper (0.1)	2085.33***	−562.03	[3.71]

***p < 0.01. All estimates are statistically significant at the 1% level.

### Urban-rural disparities

Our urban-rural heterogeneity analysis reveals that the effect of health insurance is 62% larger in urban areas than in rural areas. This disparity likely reflects the fundamental differences in benefit design between the insurance schemes dominant in each region. The Urban Employee Basic Medical Insurance (UEBMI), with its higher reimbursement rates and broader coverage, appears to enable greater medical expenditure at the end of life compared to the New Rural Cooperative Medical Scheme (NCMS). This finding underscores an important dimension of inequality in China’s healthcare system and suggests that strengthening the benefit package of NCMS could be a key policy lever for reducing urban-rural disparities in end-of-life care.

The regional analysis revealed significant spatial heterogeneity in the effect of health insurance on medical expenditures. The effect was strongest in the Eastern region (ATT = 2985 RMB, *p* < 0.01), followed by the Western region (ATT = 1858 RMB, *p* < 0.05). The effect was not statistically significant in the Central region, and no significant effect was found in the Northeastern region. This gradient pattern aligns closely with the distribution of healthcare resources across China.

Contrary to the raw association, our PSM analysis reveals that health insurance has no significant causal effect on nursing care costs, even for the most vulnerable subgroup of severely disabled elderly. The consistent negative point estimate after matching suggests that, if anything, insurance might slightly reduce out-of-pocket nursing costs—possibly by freeing up family resources through coverage of medical expenses—though this effect is not statistically significant. This finding underscores that insurance does not drive increased utilization or spending on long-term custodial care, which remains predominantly financed through out-of-pocket and family payments.

The subgroup analyses revealed a consistent pattern: in the unmatched samples, health insurance was associated with higher nursing care costs (overall sample: +1826 RMB, *p* < 0.01). However, after controlling for confounders via matching, this relationship reversed to null or negative in all subgroups (overall sample: −914 RMB, *p* = 0.14). This pattern indicates that the initial positive association was primarily driven by selection bias and that health insurance has no significant causal effect on nursing care costs.

## Discussion

In summary, our research has thoroughly analyzed the influence of health insurance on the healthcare expenses incurred by elderly Chinese citizens during their final year of life. Using PSM to mitigate potential selection bias of insurance enrollment, we have uncovered a substantial disparity in medical expenditures between individuals who are insured and those who are uninsured.

Nevertheless, our analysis reveals that health insurance has no significant effect on nursing care costs, even for the most disabled elderly. This finding is empirically supported by payment source data, which show that insurance serves as the primary payer in only 2.1% of cases, while the overwhelming cases still rely on out-of-pocket and family payments (over 90% combined). Our comprehensive subgroup analyses also demonstrate that the null effects of health insurance on nursing care costs are remarkably consistent across all population subgroups. Such consistent null findings across diverse populations strongly indicate that health insurance does not drive nursing care utilization in China’s current system, highlighting a structural gap in long-term care coverage.

In terms of factors that influence the total healthcare spending, we have found that older adults living in urban settings, cohabiting with a partner, and with good pain management tend to exhibit more substantial impacts on the upper ranges of healthcare expenditure. Conversely, income appears to have a relatively minor influence. It is worth noting that gender does not significantly affect healthcare expenditure in most cases. Furthermore, health status has emerged as a crucial determinant of medical service expenditure. In particular, elderly individuals experiencing severe pain and demonstrating lower ADL scores tend to incur greater healthcare costs.

Our findings also reveal the significant urban-rural disparity in insurance effects, which underscores structural inequalities that favor urban residents in China’s health system. This pattern aligns with the institutional design of different insurance schemes in which UEBMI and Urban Resident Basic Medical Insurance (URBMI) offer much higher reimbursement rates and broader service coverage than NCMS. Policy efforts to reduce healthcare disparities should focus on strengthening the benefit package of NCMS to narrow this gap.

Our analysis of regional heterogeneity reveals that the effect of health insurance on medical expenditures varies dramatically across China, with the strongest impact in the most developed eastern region (2985 RMB, *p* < 0.01) and more modest effects in central and western regions (approximately 1800 RMB). This spatial pattern reflects the interplay between insurance coverage and regional healthcare supply: even with insurance, individuals in less developed regions face constraints in accessing care due to supply factors, such as limited hospital beds and lower reimbursement rates. The null effect in the northeastern region also warrants further investigation into the structural barriers that may undermine insurance effectiveness. These findings highlight that achieving equitable health financial protection requires not only expanding insurance coverage but also addressing fundamental regional disparities in health systems.

In sum, our study attempts to address several end-of-life challenges confronting heatlh systems, including persistent disparities in insurance coverage and mounting pressure on cost containment, and makes at least three main contributions. Firstly, we observed a spending gap between insured and uninsured individuals, highlighting the persisting inequality inherent in China’s health system, given that insurance enrollment in China is frequently tied to place of residence and employment status. These institutional features exacerbate inequities by widening disparities in end-of-life medical spending. Secondly, by examining the impact of population aging on healthcare, our study presents novel evidence on the relationship between proximity to death and end-of-life healthcare expenditure, which contributes to the ongoing discussion surrounding “red-herring hypothesis”. Lastly, another added value of our study is to provide new evidence using data from China, where culturally and institutionally distinct conceptions of a “good death” may shape end-of-life spending patterns in ways that vastly differ from those observed in Western countries.

Our study also opens multiple avenues for future research. With more detailed data, there are at least three promising directions of subsequent studies. First, future research can delve deeper into how insurance coverage and healthcare expenditure might potentially improve health outcomes, such as pain relief, at the end of life. Secondly, future studies can explore the variations in decision-making processes, such as the use of excessive and futile treatment nearing the end of life, among insured and uninsured individuals. Lastly, future research could benefit from data that distinguish among China’s major health insurance schemes, such as UEBMI, URBMI, and NCMS. Examining their differences would offer more nuanced insights into the design of equitable health systems.

## Methods

This study used secondary data from the Chinese Longitudinal Healthy Longevity Survey (CLHLS). CLHLS is the largest longitudinal survey on the health and longevity of older adults in China, and has the largest sample of centenarians in the world^30,31^. The project focuses on individuals aged 80 and above, comprising a nationally representative sample spanning 22 provinces in China. CLHLS gathers comprehensive information on respondents and their primary caregiver, including details on health insurance coverage and healthcare service utilization. For our analysis, there were 2226 recorded deaths between 2014 and 2018, and 8668 between 2005 and 2014. Consequently, our study sample encompassed a total of 10,894 decedents. The data were accessed for research purposes on June 30, 2021. All data were fully anonymized before we accessed them. The authors have no access to information that could identify individual participants during or after data collection.

### Dependent variables

**Fig. 2:**
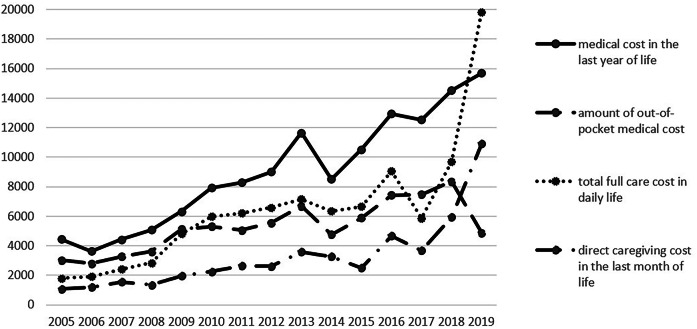
Changing trends in healthcare expenditures by types (*N* = 8839). This figure shows trends in healthcare-related expenditures from 2005 to 2019 by type of spending. Medical costs incurred in the last year of life are shown as a solid line with circular markers. Out-of-pocket medical costs are shown as a dashed line with circular markers. Total full care costs in daily life are shown as a dotted line with star markers. Direct caregiving costs incurred in the last month of life are shown as a dashed line with solid circular markers. All expenditures are measured in RMB and averaged across individuals in the sample.

We investigated healthcare expenses in two distinct areas during the final year of life: medical services and nursing care. The cost of medical services was ascertained by inquiring, “What was the total medical expenditure for the elderly individual in their last year of life?” Additionally, we recorded out-of-pocket payments by asking, “What portion of the expenses was self-funded?” Likewise, the total expenses for nursing care were obtained from inquiries such as “What were the overall nursing care costs?” and “What was the direct expense for nursing care in the final month of life?” Both above two variables are reported in Fig. 2.

### Independent variables

The focal variable pertains to whether patients relied on health insurance as their primary payment method, with the metric derived from the query “What was the primary source of payment for the deceased?” We categorized the “insured” cohort as those individuals who utilized health insurance (comprising “national medical fund,” “state or collective subsidy,” “cooperative medical fund,” or “medical insurance”) as their primary payment source for medical costs. The “uninsured” designation encompassed the respondents who did not fall within this insured group.

In this dataset, the insurance category reflects a broad grouping of China’s insurance programs rather than specific schemes such as the UEBMI, URBMI, and the NCMS. As such, our measure captures the overall view of insured versus uninsured status without distinguishing among these program types.

### Control variables

In addition to the health insurance coverage, numerous factors can affect healthcare expenditure^32^. To account for demographic influences like gender, age, and marital status, we incorporated these variables to determine their impact on medical expenditure^33,34^. We also incorporated place of residence (urban vs. rural), educational attainment, and annual household incomes into our analysis. Moreover, we adjusted for health-related aspects such as quality of life and self-assessed health status^35^. Specifically, we constructed a quality-of-life index by summing six daily activity scores. The quality-of-life index ranged from 6 to 18 as each component had three scales (1 = fully competent; 2 = partially competent; 3 = fully incompetent). Self-perceived health status was determined by the question “Did the deceased elder experience pain before death?”. Additionally, we included the site of death (home or hospital) in our models, a variable unique to end-of-life studies, which was deemed significant^36,37^.

To explore potential heterogeneous effects of health insurance, we conducted subgroup analyses by urban-rural residence. This approach serves as a proxy for analyzing different insurance schemes, as China’s UEBMI and URBMI primarily cover urban residents, while the NCMS exclusively covers rural residents.

To examine regional heterogeneity, we classified the sample into four major economic regions—Eastern, Central, Western, and Northeastern China—according to the standard classification of the National Bureau of Statistics. PSM analysis was conducted separately for each region.

To examine the heterogeneity in the effect of health insurance on nursing care costs, we conducted subgroup analyses based on functional disability status (severely disabled vs. others) and urban-rural residence. All analyses employed the same PSM method as the primary analysis (1:1 nearest neighbor matching with a caliper of 0.05).

### Descriptive statistics

**Fig. 3:**
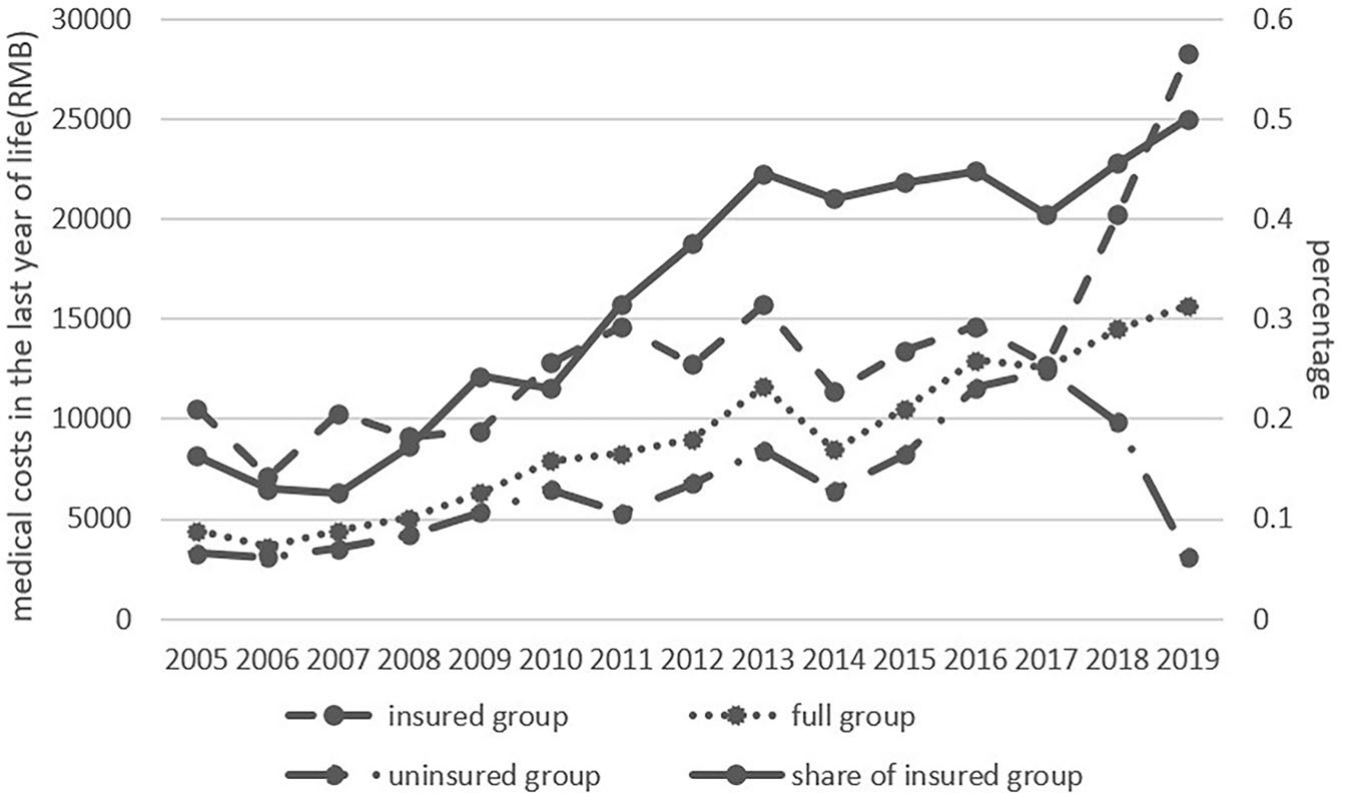
Comparison of healthcare expenditure between the insured and uninsured groups. This figure presents trends in medical costs in the last year of life for insured and uninsured individuals from 2005 to 2019. Medical expenditures (measured in RMB) for the insured group and uninsured group are plotted on the left y-axis, while the share of insured individuals in the sample is plotted on the right y-axis as a percentage. The dotted line represents the full sample average medical costs, the dashed line represents the insured group, and the solid line represents the uninsured group. The solid line with circular markers corresponds to the share of the insured group.

Table 7 depicts the descriptive statistics of all variables. Overall, only 23.8% of the deceased used health insurance to pay their medical costs. Figures 2 and 3 summarize the yearly trends of different types of medical spending for the decedents. Figure 2 illustrates a consistent rise in healthcare expenses over the last 15 years. Figure 3 shows that individuals with health insurance consistently incur higher medical service costs compared to those without coverage. The disparity between the insured and uninsured cohorts has progressively widened since 2017.

**Table 7:** Summary statistics of the decedents

Variable names	Definitions	Full sample (*N* = 8339)		Insurance paying most (*N* = 1986)		Family paying most (*N* = 6353)	
		*N*	Proportions (%)	*N*	Proportions (%)	*N*	Proportions (%)
*Inspay*	*Main source of payment*						
1	The health insurance	1986	23.82	1986	100		
0	Others	6353	76.18			6353	100
*Demographic characteristics*							
*Age*	*Age*						
[65, 70]	<70	63	0.76	13	0.65	50	0.79
[70, 79]	70–79	686	8.23	222	11.18	464	7.30
[80, 89]	80–89	1652	19.81	496	24.97	1156	18.20
[90, 99]	90–99	3257	39.06	756	38.07	2501	39.37
[100, 109]	100–109	2544	30.51	470	23.67	2074	32.65
[110, 121]	≥110	137	1.64	29	1.46	108	1.70
*Female*	*Gender*						
0	Male	3499	41.96	1086	54.68	2413	37.98
1	Female	4840	58.04	900	45.32	3940	62.02
*City*	*place of residence*						
1	Urban (city or town)	3494	41.90	1170	58.91	2324	36.58
0	Rural (village)	4845	58.10	816	41.09	4029	63.42
Cohabit	Marital Status						
1	Married and live with the spouse^a^	1456	17.46	508	25.58	948	14.92
0	Others^b^	6883	82.54	1478	74.42	5405	85.08
*Socioeconomic Characteristics*							
*Schooling*	*Education*						
0	Illiterate	5665	67.93	1077	54.23	4588	72.22
1	1–6 years of schooling^c^	2101	25.19	626	31.52	1475	23.22
2	7–9 years of schooling^d^	307	3.68	135	6.80	172	2.71
3	10–12 years of schooling^e^	170	2.04	79	3.98	91	1.43
4	>13 years of schooling^f^	96	1.15	69	3.47	27	0.42
*Income*	*Annual family income*	31,565.68		46,524.38		26,889.47	
*Health Condition*							
*Qolind*	*Quality of life index*						
6	6	1443	17.30	360	18.13	1,083	17.05
7	7	329	3.95	79	3.98	250	3.94
8	8	222	2.66	53	2.67	169	2.66
9	9	194	2.33	36	1.81	158	2.49
10	10	311	3.73	68	3.42	243	3.82
11	11	479	5.74	99	4.98	380	5.98
12	12	507	6.08	100	5.04	407	6.41
13	13	279	3.35	70	3.52	209	3.29
14	14	421	5.05	103	5.19	318	5.01
15	15	461	5.53	115	5.79	346	5.45
16	16	1,012	12.14	240	12.08	772	12.15
17	17	848	10.17	224	11.28	624	9.82
18	18	1833	21.98	439	22.10	1,394	21.94
*Pain*	*Painfulness*						
1	Very painful	323	3.87	118	5.94	205	3.23
2	Painful	882	10.58	247	12.44	635	10.00
3	Average	2093	25.10	518	26.08	1575	24.79
4	Relatively peaceful	3196	38.33	692	34.84	2504	39.41
5	Peaceful	1845	22.12	411	20.69	1434	22.57
*Dhospital*	*Place of death*						
1	Hospital	751	9.01	399	20.09	352	5.54
0	Others^g^	7588	90.99	1587	79.91	6001	94.46

^a^Including the cohabiting partners.

^b^Including married but not living with the spouse, divorced, widowed, and never married.

^c^Equivalent of elementary school.

^d^Equivalent of junior high school.

^e^Equivalent of senior high school.

^f^Equivalent of having a bachelor’s degree or above.

^g^Including home, institutions and others.

### Empirical methods

To assess the influence of health insurance on end-of-life expenditure, we employed a range of models, including OLS, quantile regression, Tobit, and PSM. The quantile regression model evaluates effects across various quantiles of the outcome variables. Additionally, we utilized the Tobit model due to its effectiveness in handling zero values and censored income data. Subsequently, we utilized propensity scores to match insured and uninsured individuals based on observable factors. Our baseline model is specified as follows:

1 $${{y}}_{{i},{t}}={{\beta }}_{0,{t}}+{{\beta }}_{1,{t}}{\rm{inspay}}+{\rm{{\rm B}}}{{X}}_{{i},{t}}+{{\lambda }}_{{t}}+{{\delta }}_{{j}}+{{\varepsilon }}_{{i},{t}}$$

where *y*_*i,t*_ is the medical costs towards the end of life of individual *i* in year *t* of the survey; *inspay* is the main variable of interest and a dummy variable identifying whether one belongs to the insured group (*inspay* = 1 if health insurance was the main financial source of medical payment; 0 otherwise); *X*_*i,t*_ denotes a vector encompassing control variables such as education, gender, age, marital status, and income; ${\varepsilon }_{i.t}$ is an error term. To account for the rising trend in medical expenditure over time, we included year fixed effects ${\lambda }_{t}$. Province fixed effects ${\delta }_{j}$ are also added to capture the provincial variations in institutional factors, such as regional policies.

We also tried to address the selection bias in terms of having access to health insurance. Patients who predominantly relied on health insurance for payment may differ systematically from those who did not. Propensity score matching equalizes the probability of receiving treatment between insured and uninsured groups, thereby creating a comparison group that serves as a counterfactual. Propensity scores are estimated using observable factors that might influence treatment assignment.

In our model, we employed Logistic regression to estimate propensity scores, incorporating demographic characteristics, socioeconomic factors, health conditions, and year fixed effects. We adopted a one-to-one nearest neighbor matching approach with a 0.05 caliper to identify the corresponding comparison group. Samples outside the common support region were excluded. We ascertained the average treatment effect on the treated by calculating the difference in expenditure between older adults whose costs were primarily paid by health insurance ($inspa{y}_{i,t}=1$) and those whose costs were mainly paid by other sources.

## Supplementary information

Supplementary information

## Data Availability

The data of this study are accessible from https://opendata.pku.edu.cn/dataverse/CHADS, 10.18170/DVN/WBO7LK. Restrictions may apply to the availability of these data. However, data can be obtained from the authors upon reasonable requests and with permission from the PKU Center for Healthy Aging and Development (chads@nsd.pku.edu.cn).
