# Supplementary material for: Health insurance and end-of-life healthcare expenditures: evidence from Chinese Longitudinal Healthy Longevity Survey
Source: Npj Health Syst. 2026 Apr 27;3:26. doi: 10.1038/s44401-026-00084-1 (PMC13354233; doi:10.1038/s44401-026-00084-1)
Supplement: Supplementary file 1 — Supplementary information [file 44401_2026_84_MOESM1_ESM.pdf]

Supplementary Table 1

Heterogeneous effects of health insurance on medical expenditures by place of residence

Subgroup	ATT (RMB)	(S.E.)	[t-stat]	Matched Sample (Treated/Control)	Total Matched
Urban	3057.93***	-820.83	[3.73]	974 / 2,324	3298
Rural	1889.21***	-710.07	[2.66]	805 / 4,029	4834

Supplementary Table 2

Heterogeneous effects of health insurance on medical expenditures by geographical region

Region	ATT (RMB)	(S.E.)	[t-stat]	Matched Sample
Eastern	2984.63***	-1004.17	[2.97]	748/2,236
Central	1791.97	-1121.96	[1.60]	423/1,726
Western	1858.07**	-899.82	[2.06]	492/1,933
Northeastern	-440.55	-2010.26	[-0.22]	120/458

***p<0.01, **p<0.05*

Supplementary Table 3

Heterogeneous effects of health insurance on nursing care costs

Subgroup	Unmatched Difference	ATT (Matched)	(S.E.)	[t-stat]	p-value	Matched Sample (Treated/Control)
Overall Sample	1,826.24***	-914.28	-614.67	[-1.49]	0.14	1,748/6,308
By Functional Status						
∟ Severely Disabled	2,243.12*	-1838.1	-1373.77	[-1.34]	0.18	387/1,388
∟ Other Elderly	1,690.02***	-1156.37	-699.49	[-1.65]	0.1	1,352/4,920
By Residence						
∟ Urban	1,527.71*	-1542.71	-865.43	[-1.78]	0.075	958/2,310
∟ Rural	1,299.36*	-39.99	-853.77	[-0.05]	0.96	775/3,998

Supplementary Table 4

Primary Payer for Nursing Care Costs (%)

Payment Source	Overall Sample (N=8,043)	Insured Group (N=1,862)	Uninsured Group (N=6,181)
Medical Insurance	0.0239	0.0945	0.0026
Out-of-Pocket (Self)	0.0556	0.131	0.0328
Spouse	0.0117	0.015	0.0107
Family (Children/Grandchildren)	0.7579	0.5704	0.8144
State/Collectives	0.0155	0.0612	0.0018
Others	0.0179	0.0317	0.0138
